# Polish Adaptation of the Basic Psychological Need Satisfaction and Frustration Scale

**DOI:** 10.3389/fpsyg.2019.03034

**Published:** 2020-01-29

**Authors:** Beata Kuźma, Michał Szulawski, Maarten Vansteenkiste, Katarzyna Cantarero

**Affiliations:** ^1^Faculty of Psychology and Law, SWPS University of Social Sciences and Humanities, Poznań, Poland; ^2^Institute of Psychology, The Maria Grzegorzewska University, Warsaw, Poland; ^3^Department of Developmental, Personality and Social Psychology, Ghent University, Ghent, Belgium; ^4^Social Behavior Research Center, SWPS University of Social Science and Humanities, Wrocław, Poland

**Keywords:** basic psychological needs, Self-Determination Theory, well-being, depressive symptoms, Basic Psychological Need Satisfaction and Frustration Scale

## Abstract

This article presents the findings of four studies designed to validate the translated Polish version of the Basic Psychological Need Satisfaction and Frustration Scale. Results of exploratory factor analyses in Study 1 (*N* = 272, *M*_age_ = 41.07) showed that the psychological need for autonomy, relatedness, and competence that are central to the Self-Determination Theory have a bidimensional structure, involving both a need for satisfaction and need for frustration component. Subsequent confirmatory factor analyses in Study 2 (*N* = 265; *M*_age_ = 38.15) provided further evidence for a six-dimensional structure of the scale, thereby distinguishing a satisfaction and frustration component for each of the three needs. Study 3 (*N* = 158; *M*_age_ = 27.28) further revealed that the distinguished subscales are moderately to highly internally consistent and yielded good test–retest reliability. Finally, Study 4 (*N* = 204; *M*_age_ = 20.57) confirmed that satisfaction of the needs is positively related to well-being, while frustration is positively related to depressive symptoms. The Polish version of the Basic Psychological Need Satisfaction and Frustration Scale can be successfully used in future basic and applied studies in the context of Self-Determination Theory.

## Introduction

The question of which factors promote well-being and thriving has been central to Basic Psychological Need Theory, one of the six mini-theories of the Self-Determination Theory (Deci and Ryan, 2000; Vansteenkiste et al., 2010; Ryan and Deci, 2017). In addressing this question, it is argued within the Basic Psychological Need Theory that humans have a set of basic psychological needs that serve as essential nutrients for individuals’ growth, integrity, and well-being. Although the list of needs is open to additions, three have been distinguished so far, that is, autonomy, relatedness, and competence. Although dozens of studies have now demonstrated the well-being benefits associated with need satisfaction and the costs associated with need frustration, their role in Polish individuals’ functioning has been largely neglected so far. To foster research in this community, the present study, involving a series of four studies, aimed to provide formal evidence for the validity of the translated Polish version of the Basic Psychological Need Satisfaction and Frustration Scale (BPNSFS; Chen et al., 2015).

### Need Satisfaction and Need Frustration

The Basic Psychological Needs Theory describes human beings as having three basic psychological needs (autonomy, competence, and relatedness), which serve as social nutrients for developing autonomous motivation and well-being. Autonomy satisfaction occurs when one experiences a sense of volition, psychological freedom, and authorship in one’s thinking, acting, and feeling, while autonomy frustration denotes a feeling of control, pressure, and conflict. Competence involves feeling effective and capable during action, while competence frustration denotes a feeling of inadequacy and failure. Relatedness involves feeling meaningfully and warmly connected to others, while relatedness frustration denotes feelings of social alienation and loneliness (Deci and Ryan, 2000; Ryan and Deci, 2017).

Early research in the Basic Psychological Needs Theory paradigm focused primarily on the benefits of need satisfaction for individuals’ thriving. A broad variety of well-being indicators were found to be systematically related to individuals’ need satisfaction, including their vitality and energy (e.g., Gagné et al., 2003), self-esteem (Moller et al., 2006), and positive mood (Sheldon and Bettencourt, 2002; Baard et al., 2004; Ebersold et al., 2019). Such findings have been confirmed in diverse life domains, including work, sports, and education (Sheldon and Filak, 2008; Sheldon et al., 2013; Chiniara and Bentein, 2016), and in nations differing in terms of their cultural heritage and focus (e.g., Church et al., 2013; Chen et al., 2015).

Because it was increasingly recognized that low need satisfaction does not imply the presence of need frustration (Bartholomew et al., 2011; Vansteenkiste and Ryan, 2013), the study of the frustration of one’s psychological needs received increasing attention in its own right. To illustrate, although the need for relatedness may not be satisfied if a person on a given day does not feel well connected to the group of people he works with, he still may not feel excluded or rejected, which would signal the presence of relatedness need frustration (Vansteenkiste and Ryan, 2013). For need frustration to take place, a more active threat to that need is required.

Studying the separate dynamics of need satisfaction and need frustration appeared fruitful because need frustration was found to be especially predictive for the “dark” side of individuals’ functioning. That is, need frustration robustly relates to ill-being (e.g., Bartholomew et al., 2011; Stebbings et al., 2012), disengagement (e.g., Jang et al., 2016), and poor sleep (Campbell et al., 2018). These findings, which involved self-reported indicators of maladjustment, were corroborated in studies that made use of objective markers, such as athletes’ S-IgA (i.e., immunological protein in saliva; Bartholomew et al., 2011) and elevated blood pressure (Weinstein et al., 2011). A flurry of recent studies has further confirmed the multiple costs associated with need frustration beyond ill-being, as indexed by disordered eating (Verstuyf et al., 2013) as well as rigidly and obsessively pursued behaviors (Tóth-Király et al., 2019). Such findings have been established in different age groups, from adolescents (Talwar and Lee, 2011; Vandenkerckhove, 2019) to adults (Van den Broeck et al., 2016) and seniors living in nursing homes (Ferrand et al., 2019). It seems that associations with both the satisfaction and frustration of needs is universal and pertain to numerous domains.

Many of the studies addressing the role of need satisfaction and frustration have made use of the BPNSFS (Chen et al., 2015), the widely used need scale that includes a balanced combination of items from satisfaction and frustration subscales. The scale was developed on the basis of three earlier used scales, that is, (1) the Basic Psychological Need Satisfaction Scale (Ilardi et al., 1993), (2) the Balanced Measurement of Psychological Needs (Sheldon and Hilpert, 2012), and (3) Relationship Need Satisfaction Scale (La Guardia et al., 2000; La Guardia and Patrick, 2008). Different from prior studies, the BPNSFS was formally validated in four different cultures, that is, Peru, China, Belgium, and the United States. The scale consists of 24 items, 8 for each need (i.e., four for satisfaction and four for frustration) such that six subscales are distinguished. The reliability of the six subscales appeared satisfying to good in each of the four included samples, and, importantly, the obtained pattern of findings with university students’ well-being (i.e., life satisfaction, vitality) and ill-being (i.e., depressive symptoms) appeared to be invariant across the four studied countries (Chen et al., 2015). These findings speak to the universal role of the needs and, since their publication in 2015, have been replicated in dozens of different countries, with various translations and adaptations being developed (see Vansteenkiste et al., in press). To illustrate, the scale has been validated in Japanese (Nishimura and Suzuki, 2016), Portuguese (Cordeiro et al., 2016a, b), Italian (Costa et al., 2017), and Hebrew (Benita et al., 2019) to name a few translations.

### Present Research

Although the BPNSFS is now widely used by researchers around the world, it is still not available in the Polish context. There is no other tool available for measuring the basic psychological needs either. Central European countries have been underrepresented in research on basic psychological needs. We hope that the availability of a valid tool to measure BPNSFS in Polish language will foster cross-cultural research, in which Poland is also included. Our studies facilitate access to a much-needed instrument measuring psychological needs to researchers who wish to conduct basic and applied studies in the Polish context. Therefore, this project aimed to validate the scale in the Polish language. The validation consisted of several steps: First, we conducted a full language adaptation, using a parallel blind technique and blind back-translation to check accuracy (Behling and Law, 2000). Next, we conducted a series of four studies to check the dimensional structure of the Polish scale (Study 1), verify and compare the structure of the Polish scale with the original scale (Study 2), examine the test–retest reliability of the scale (Study 3), and investigate associations of the scale with theoretically related constructs – life satisfaction and depressive symptoms (Study 4). Our assumption was that the Polish scale would have similar psychometric characteristics to the original scale.

## Materials and Methods

We conducted studies to adapt the BPNSFS (Chen et al., 2015) to Polish culture. First, the items of the scale were translated into the Polish language by a bilingual translator, and then, another bilingual person back-translated the scale. We then consulted the translated English version of the scale with the authors of the original scale, and whenever inconsistencies were found, we continued the procedure until a consensus was reached that the translation was the best representation of the meaning of the items in Polish. The Polish version of the BPNSFS was then administered in various studies to test its factorial structure as well as its convergent and divergent validity. The studies were approved by the faculty’s Research Ethics Committee and were conducted in accordance with the Helsinki Declaration.

### Study 1

The aim of Study 1 was to test the factorial structure of the Polish version of the BPNSFS. To examine possible alterations to the Scale that could have taken place in the process of translation and language adaptation, we conducted an exploratory factor analysis to uncover the underlying dimensional structure of the scale.

#### Participants and Recruitment

While de Winter et al. (2009) argue that an exploratory factor analysis can be conducted with sample sizes of 50 participants, Mundfrom et al. (2005) recommend that the minimum number of participants should equal 100 and range from 3 to 20 times the number of items. Because the scale has 24 items, we aimed to collect at least 240 participants. Participants were members of a Polish research portal, who receive points for taking part in surveys, and the points can later be exchanged for small material goods (such as headphones, pen drives, etc.). Two hundred seventy-two participants (172 women, 95 men, 5 participants did not state what their gender was) took part in the study. The age of the participants ranged between 18 and 90 (*M*_age_ = 41.07, SD_age_ = 15.07). Five participants did not state what their nationality was, one participant was Ukrainian, one was Lithuanian, and the rest of the participants declared that their nationality was Polish.

#### Procedure and Materials

After giving their informed consent, participants filled in the 24-item Polish version of the Basic Need Satisfaction and Frustration Scale by evaluating how accurately each statement describes experiences and/or emotions at a given moment in their life using a 5-point Likert scale (1 = *not at all*, 5 = *very well*). We then gathered demographic data.

#### Results and Discussion

We adopted a similar approach toward data analysis of the exploratory factor analysis as did Chen et al. (2015), the authors of the original version of the scale. We performed an exploratory factor analysis on the basis of the principal axis method with Varimax rotation. Among the eight autonomy items, two factors emerged based on the eigenvalues (eigenvalues = 2.13 and 2.09) and scree plot analysis. The two factors explained 52.73% of variance. Similarly, within the eight relatedness items, there were two factors with eigenvalues above 1 (eigenvalue = 2.60 and 2.41) that explained 62.59% of variance. Finally, also among the eight competence items, two factors emerged (eigenvalue = 2.51 and 2.50) that explained 62.56% of variance. The factor loadings of the items are presented in Table 1. The Cronbach’s α of the six needs were above 0.80, indicating high reliability of the six dimensions (Table 2). Satisfaction of the needs of autonomy, relatedness, and competence were all positively and moderately related to one another. Similarly, the scales of frustration of the three needs were also moderately and positively related. As in Chen et al. (2015), satisfaction and frustration of the needs were negatively related. These findings give initial support in that the three psychological needs have a two-dimensional structure of need satisfaction and need frustration.

**TABLE 1:** Factor loadings based on exploratory factor analysis for 24 items of the Basic Need Satisfaction and Frustration Scale (*N* = 272, Study 1).

	Autonomy		Relatedness		Competence	
			
	Satisfaction	Frustration	Satisfaction	Frustration	Satisfaction	Frustration
1. I feel that my decisions reflect what I really want	0.84					
2. I feel I have been doing what really interests me	0.67					
3. I feel my choices express who I really am	0.66					
4. I feel a sense of choice and freedom in the things I undertake	0.60					
5. I feel forced to do many things I wouldn’t choose to do		0.76				
6. I feel pressured to do too many things		0.74				
7. Most of the things I do feel like “I have to”		0.73				
8. My daily activities feel like a chain of obligations		0.63				
9. I feel close and connected with other people who are important to me			0.89			
10. I feel connected with people who care for me, and for whom I care			0.83			
11. I experience a warm feeling with the people I spend time with			0.72			
12. I feel that the people I care about also care about me			0.65			
13. I feel that people who are important to me are cold and distant toward me				0.79		
14. I feel excluded from the group I want to belong to				0.75		
15. I have the impression that people I spend time with dislike me				0.73		
16. I feel the relationships I have are just superficial				0.70		
17. I feel competent to achieve my goals					0.83	
18. I feel I can successfully complete difficult tasks					0.81	
19. I feel capable at what I do					0.76	
20. I feel confident that I can do things well					0.62	
21. I feel like a failure because of the mistakes I make						0.82
22. I feel insecure about my abilities						0.77
23. I have serious doubts about whether I can do things well						0.75
24. I feel disappointed with many of my performance						0.68

*All factor loadings below 0.50 are suppressed.*

**TABLE 2:** Means, standard deviations, reliabilities, and correlations of the Basic Psychological Needs, Study 1.

	*M*	SD	1	2	3	4	5	6
**Satisfaction**								
1. Autonomy	3.67	0.78	α = 0.80					
2. Relatedness	3.93	0.88	0.63**	α = 0.82				
3. Competence	3.90	0.77	0.73**	0.63**	α = 0.87			
**Frustration**								
4. Autonomy	2.74	0.94	−0.34**	−0.19*	−0.27**	α = 0.85		
5. Relatedness	2.18	0.93	−0.33**	−0.45**	−0.41**	0.69**	α = 0.87	
6. Competence	2.32	1.01	−0.40**	−0.29**	−0.48**	0.70**	0.76**	α = 0.87

*N* = 272, *p < 0.01, **p < 0.001. Reliabilities are written in diagonal.

### Study 2

In Study 2, we tested the robustness of the results obtained in Study 1. We conducted a confirmatory factor analysis verifying the six-dimensional structure of the BPNSFS.

#### Participants and Recruitment

Given the recommendations of Mundfrom et al. (2005), we wanted to conduct the study on as many participants as possible within a set period of time. Participants were 265 Polish respondents, members of a research portal who completed an online study (167 women, 92 men, 6 individuals did not state what their gender was). The age of the students ranged between 18 and 90 (*M*_age_ = 38.15, SD_age_ = 14.07). Participants received points for participation that can be exchanged for small material goods.

#### Procedure and Materials

After giving their informed consent, participants completed the 24-item BPNSFS. We then gathered demographic data.

#### Results and Discussion

We tested whether the data corresponded to the six-factorial model. A generalized least squares confirmatory factor analysis yielded good fit, χ^2^ = 399.34, df = 237, χ^2^/df = 1.69; root mean square error of approximation = 0.052, 90% CI = [0.043, 0.069], *p* = 0.378 and adjusted goodness-of-fit index = 0.84.

We also analyzed a three-factorial solution (autonomy, relatedness, and competence) with frustration and satisfaction items of each of the need joints. Although such a model fitted the data well, it had a worse fit than the six-factorial solution, χ^2^/df = 1.97; root mean square error of approximation = 0.061, 90% CI = [0.053, 0.069], *p* = 0.010. In addition, the Akaike information criterion (AIC) measure of fit was lower in the case of the six-factorial model, AIC = 525.34, than the three-factorial model AIC = 593.50, which bolstered our trust that the six-factorial model should be retained. All of the standardized regression weights were above 0.50 (Table 3, for Polish version see Appendix 1).

**TABLE 3:** Standardized regression weighs in confirmatory factor analysis (CFA) of the 24 items of the Basic Need Satisfaction and Frustration Scale (*N* = 259, Study 2).

	Autonomy		Relatedness		Competence	
			
	Satisfaction	Frustration	Satisfaction	Frustration	Satisfaction	Frustration
I feel a sense of choice and freedom in the things I undertake	0.56					
Most of the things I do feel like “I have to”		0.57				
I feel that people I care about also care about me			0.78			
I feel excluded from the group I want to belong to				0.76		
I feel confident I can do things well						
I have serious doubts about whether I can do things well					0.72	
I feel that my decisions reflect what I really want	0.69					0.83
I feel forced to do many things I wouldn’t choose to do		0.77				
I feel connected with people who care for me, and for whom I care			0.76			
I feel that people who are important to me are cold and distant toward me				0.79		
I feel capable at what I do					0.78	
I feel disappointed with many of my performance						0.70
I feel my choices express who I really am	0.74					
I feel pressured to do too many things		0.69				
I feel close and connected with other people who are important to me			0.80			
I have the impression that people I spend time with dislike me				0.77		
I feel competent to achieve my goals					0.81	
I feel insecure about my abilities						0.77
I feel I have been doing what really interests me	0.66					
My daily activities feel like a chain of obligations		0.63				
I experience a warm feeling with the people I spend time with			0.78			
I feel the relationships I have are just superficial				0.72		
I feel I can successfully complete difficult tasks					0.80	
I feel like a failure because of mistakes I make						0.79

*This was the order of the items as it was presented to the participants.*

In addition, we analyzed the internal consistency of the scales in this sample. The results were as follows: autonomy satisfaction scale α = 0.72, autonomy frustration α = 0.75, relatedness satisfaction α = 0.83, relatedness frustration α = 0.81, competence satisfaction α = 0.83, competence frustration α = 0.81. These results confirm the six-dimensional structure of the BPNSFS.

### Study 3

The aim of Study 3 was to analyze test–retest reliability of the BPNSFS. Specifically, we tested if people’s scores on the BPNSFS were relatively stable over time.

#### Participants and Procedure

We gathered data from 158 Polish students (135 women) *M*_age_ = 27.28, SD = 8.49 who filled in the BPNSFS twice with a break of 4 weeks between the two measurements (first measurement at T1 and second measurement at T2). The minimum break was 20 days, and the maximum was 45 days with a mean value of 30 days and a standard deviation of 4 days. Participation was rewarded with course credit points. We asked participants to complete the BPNSFS and to provide demographic data. The overall sample size yields corresponding power in excess of (1 − β) = 0.95, for lower and upper critical *r* of 0.37 (α = 0.05, one-tailed).

#### Results and Discussion

Internal consistency of the subscales measured with Cronbach’s α for both measurements was high and was, respectively, for T1 and T2: autonomy satisfaction, 0.79 and 0.84; autonomy frustration, 0.79 and 0.82; for relatedness satisfaction, 0.75 and 0.83; relatedness frustration, 0.77 and 0.76; and finally, for competence satisfaction, 0.87 and 0.89 and for competence frustration, 0.87 and 0.86. The test–retest reliability of the subscales was moderate with correlations between T1 and T2 ranging from *r* = 0.63 (relatedness satisfaction) to *r* = 0.79 (competence frustration) (*p* < 0.001). These results indicate good test–retest reliability of the BPNSF Scale. In addition, to check the differences between T1 and T2 measures, we have conducted paired sampled *t* test for all six subscales. There were no significant differences between autonomy satisfaction at T1 (*M* = 3.87, SD = 0.79) and autonomy satisfaction at T2 (*M* = 3.81, SD = 0.82), *t*_157_ = 1.20, *p* = 0.231, *d* = 0.1; between autonomy frustration at T1 (*M* = 2.63, SD = 0.86) and autonomy frustration at T2 (*M* = 2.68, SD = 0.92), *t*_157_ = −0.92, *p* = 0.359, *d* = 0.07; between relatedness satisfaction at T1 (*M* = 4.29, SD = 0.59) and relatedness satisfaction at T2 (*M* = 4.22, SD = 0.70), *t*_157_ = 1.69, *p* = 0.092, *d* = 0.14; between relatedness frustration at T1 (*M* = 1.83, SD = 0.73) and relatedness frustration at T2 (*M* = 1.85, SD = 0.71), *t*_157_ = −0.60, *p* = 0.551, *d* = 0.05); between competence satisfaction at T1 (*M* = 3.89, SD = 0.78) and competence satisfaction at T2 (*M* = 3.87, SD = 0.80), *t*_157_ = 0.42, *p* = 0.677, *d* = 0.03; and between competence frustration at T1 (*M* = 2.24, SD = 0.95) and competence frustration at T2 (*M* = 2.30, SD = 0.97), *t*_157_ = −1.06, *p* = 0.290, *d* = 0.08). These results indicate good time invariance of the BPNSFS.

### Study 4

Thus far, we had focused on the internal structure of the BPNSFS. In Study 4, we examined the convergent validity of the scale using the Multitrait–Multimethod Matrix (Campbell and Fiske, 1959). The method measures convergent and discriminant validity by comparing how the constructs from the particular scale relate to similar and different constructs measured by other scale. In our study, we anticipated moderate associations between the satisfaction and frustration of the basic psychological needs and satisfaction with life and depressive symptoms, demonstrating their relatedness but not interchangeableness. More specifically, as in the study by Chen et al. (2015), we expected that satisfaction of the needs would be positively related to satisfaction with life, and frustration of the needs would be negatively correlated with it. We anticipated that satisfaction of the basic psychological needs would be negatively related to depressive symptoms and frustration of the needs would be positively related to them.

#### Participants and Recruitment

We aimed at maximizing the number of participants we could reach within the time period allotted to the study. Two hundred four students (169 women, 35 men) took part in a paper-and-pencil study. Ages ranged from 19 to 24 (*M*_age_ = 20.57, SD_age_ = 1.26). Participation in the study was compensated with course credit points. We calculated achieved power for the lowest correlation between the variables of interest in the study. The overall sample size yields corresponding power in excess of (1 – β) = 0.95 for *r* value of 0.37 (α = 0.05, two-tailed) with lower and upper critical *r* = 0.14.

#### Procedure and Materials

We then asked participants to fill in three scales: the five-item Polish version of the Satisfaction with Life Scale (α = 0.81, Diener et al., 1985; Juczyński, 2001; e.g., “*In most ways my life is close to my ideal*,” 1 = *strongly disagree*, 7 = *strongly agree*); the 24-item Polish version of the BPNSFS, and the 20-item Polish version of the Center for Epidemiologic Studies Depression Scale (CES-D, α = 0.90, Radloff, 1977; Jankowski, 2016). The order of the scales was fixed and presented to the participants in the previously mentioned order (Satisfaction with Life Scale, BPNSFS, CES-D).

#### Results and Discussion

First, we recoded items that were reverse scored in CES-D. We then computed mean replies of the scale so that higher number indicated higher intensity of a dimension (satisfaction of life, etc.). The Cronbach’s α of the BPNSFS subscales in this study were as follows: autonomy satisfaction α = 0.62, autonomy frustration α = 0.71, relatedness satisfaction α = 0.78, relatedness frustration α = 0.78, competence satisfaction α = 0.82, and competence frustration α = 0.75. Satisfaction of the three types of basic needs was positively related to satisfaction with life and negatively with CES-D. These results are displayed in Table 4.

**TABLE 4:** Correlations between basic psychological needs, satisfaction with life and depressive symptoms.

	1	2	3	4	5	6	7	8
**Satisfaction**								
1. Autonomy	α = 0.62							
2. Relatedness	0.56*	α = 0.78						
3. Competence	0.54*	0.53*	α = 0.82					
**Frustration**								
4. Autonomy	−0.33*	−0.37*	−0.43*	α = 0.71				
5. Relatedness	−0.53*	−0.59*	−0.52*	0.43*	α = 0.78			
6. Competence	−0.52*	−0.35*	−0.57*	0.50*	0.57*	α = 0.75		
7. Satisfaction with life	0.47*	0.51*	0.55*	−0.40*	−0.37*	−0.47*	α = 0.81	
8. Depressive symptoms	−0.56*	−0.44*	−0.47*	0.49*	0.52*	0.58*	−0.49*	α = 0.88

*N* = 204, * *p* < 0.001.

Satisfaction of the basic psychological needs correlated positively and moderately with satisfaction with life, and it was negatively related to the frustration of the needs. Frustration of the basic psychological needs correlated positively with depressive symptoms and satisfaction of the basic psychological needs correlated with it negatively. These results corroborate those of Chen et al. (2015) and indicate the convergent validity of BPNSFS: it does relate to theoretically relevant constructs, but at the same time, the relatively modest relationship between the variables indicates that the constructs are sufficiently distinct.

## General Discussion

In this research project, we conducted four studies to adapt and validate the BPNSFS to the Polish language and culture where no such scale was available so far. The results indicate that the Polish version of the scale is a reliable measure and presents similar psychometric features to the original version (Chen et al., 2015).

Our findings corroborate previous findings indicating a six-dimensional model of the scale. The six dimensions that form basic psychological needs are autonomy satisfaction, autonomy frustration, competence satisfaction, competence frustration, relatedness satisfaction, and relatedness frustration. The internal structure of the Polish version of BPNSFS proved to be good and similar to the original version. It also became clear that satisfaction and frustration of the three basic psychological needs are negatively correlated with each another, which supports the presumption of a two-dimensional structure of satisfaction–frustration of each psychological need.

Although the needs measured by the BPNSFS are believed to be inherent, it is important to acknowledge that their satisfaction and frustration are susceptible to contextual factors (Deci and Ryan, 2000). Thus, the authors expected a rather moderate level of reliability over time. The adapted version of the scale proved to have good test–retest reliability over time with the correlation of the T1 and T2 measures ranging from 0.63 to 0.79, with the strongest correlations for competence satisfaction and frustration, and there were no significance differences between T1 and T2 results in all the subscales indicating the Scale invariance over time.

The Polish version of the BPNSFS is internally consistent. Across all presented studies, the scale yielded good to high internal consistency measured with Cronbach’s α ranging from 0.72 to 0.87 in samples drawn from the general population and 0.62 to 0.89 across studies with the student samples.

The previously explored assumption that satisfaction of basic psychological needs should be associated with well-being and their frustration with ill-being (Vansteenkiste and Ryan, 2013) was replicated in our study with the adapted version of the BPNSFS. The results of the research confirm that indicators of well-being (subjective satisfaction with life and lack of depressive symptoms) were positively correlated with satisfaction of the need for autonomy, competence, and relatedness, and indicators of ill-being (low subjective satisfaction with life and occurrence of depressive symptoms) were positively correlated with frustration of the aforementioned needs. The correlations, however, were moderate. This implies that the construct of satisfaction and frustration of the basic psychological needs is connected but not interchangeable with the concept of satisfaction with life or depressive symptoms.

## Limitations of the Study and Recommendations for Future Research

Although we checked the factorial structure, reliability, and validity of the Polish version of the BPNSFS (all of which were at least at a satisfactory level), the study has its limitations. The first is its reliance on questionnaires as research methods – with all the benefits and weaknesses that accompany self-reports. Data based on self-report are affected by participants’ self-presentation and can also be influenced simply by a lack of knowledge in the areas that are being explored (Paulhus and Vazire, 2007; McDonald, 2008). Second, the data that we collected allowed us to conduct a correlation analysis alone. However, it is worth mentioning that self-reports are considered to be an important source of knowledge that participants have about themselves and are crucial to explore, especially in research on well-being (Diener, 1984).

Another limitation of the study is that it partially relied on a student sample – especially in Study 4, the relative homogeneity of the sample is reflected by age of the participants and the developmental period they are in. Importantly, we also used a population sample to analyze the factorial structure of the scale, which elevates our confidence in the validity of the Polish version of the scale.

We think it would be beneficial for future research to explore how basic psychological needs relate to constructs focusing on personal resources important for well-being (e.g., sense of coherence; Antonovsky, 1987, 1993). This would allow a deeper understanding of the processes related to need fulfilment and its consequences for the functioning of individuals. In addition, further studies could focus on more diverse research methods (i.e., employing experimental designs and conducting research on behavior). All of the above could give additional insights into the properties of the scale and the underlying construct as an important factor for individual well-being.

In sum, the results of the study on the Polish adaptation and validation of the BPNSFS make us confident that it can be successfully used in the Polish culture as an equivalent of the original version (Chen et al., 2015). The adapted BPNSFS is a useful instrument that allows exploration of both aspects (satisfaction as well as frustration) of the three basic and universal psychological needs from the Self-Determination Theory (Deci and Ryan, 2000) in the Polish milieu.

## Data Availability Statement

The datasets generated for this study are available on request to the corresponding author.

## Ethics Statement

The studies involving human participants were reviewed and approved by the Faculty of Psychology and Law, SWPS University in Poznań Research Ethics Committee. The patients/participants provided their written informed consent to participate in this study.

## Author Contributions

BK, MS, and KC contributed and participated in the preparation of the manuscript and all research steps: designed the study, coordinated data collection, performed analyses, and wrote the manuscript. MV consulted the accuracy of the back-translated version of the BPNSFS and participated in writing the manuscript.

## Conflict of Interest

The authors declare that the research was conducted in the absence of any commercial or financial relationships that could be construed as a potential conflict of interest.

## Appendix

**TABLE 1:** Standardized regression weighs in confirmatory factor analysis (CFA) of the 24 items of the Basic Need Satisfaction and Frustration Scale in Polish (*N* = 259, Study 2).

	Autonomy		Relatedness		Competence	
			
	Satisfaction	Frustration	Satisfaction	Frustration	Satisfaction	Frustration
Mam poczucie swobody wyboru oraz wolności w realizacji czynności, których się podejmuję	0.56					
Towarzyszy mi poczucie przymusu (“Ja muszę”) przy większości spraw, które realizuję		0.57				
Mam poczucie, że ludzie, o których się troszczę, troszczą się również o mnie			0.78			
Czuję się wykluczony z grupy, do której chcę należeć				0.76		
Czuję się pewny, że potrafię wykonywać należące do mnie zadania						
Mam poważne obawy, czy potrafię robić dobrze, to co robię					0.72	
Mam poczucie, że moje decyzje odzwierciedlają to, co faktycznie chcę w życiu	0.69					0.83
Czuję się zmuszany do rzeczy, których nie chcę robić		0.77				
Czuję się związany z ludźmi, na których mi zależy			0.76			
Czuję, że ludzie, którzy są dla mnie ważni odnoszą się do mnie,,chłodno” i z dystansem				0.79		
Czuję się kompetentny/a do wykonywania tego co robię					0.78	
Czuję się rozczarowany swoją efektywnością						0.70
Mam poczucie, że moje wybory wyrażają to kim naprawdę jestem	0.74					
Czuję presję do robienia wielu rzeczy		0.69				
Czuję się blisko związany/a z ludźmi, którzy są dla mnie ważni			0.80			
Mam wrażenie, że ludzie, z którymi spędzam czas nie lubią mnie				0.77		
Czuję się kompetentny/a aby osiągać postawione sobie cele					0.81	
Czuję się niepewny/a swoich umiejętności						0.77
Mam poczucie, że zajmuję się sprawami, które naprawdę mnie interesują	0.66					
Czuję, że moje codzienne czynności są jak łańcuch zobowiązań		0.63				
Doświadczam ciepłych uczuć od ludzi, z którymi spędzam czas			0.78			
Czuję, że moje relacje z innymi są powierzchowne				0.72		
Czuję, że mogę z sukcesem podejmować się i wykonywać trudne zadania					0.80	
Mam poczucie porażki życiowej, przez błędy, które popełniam						0.79

*This was the order of the items as it was presented to the participants.*
